# Before Guillain‐Barre Syndrome Is Attributed to a CMV Infection, the Diagnosis Must Be Confirmed and Other Causes Must Be Ruled Out

**DOI:** 10.1002/kjm2.70264

**Published:** 2026-07-08

**Authors:** Josef Finsterer

**Affiliations:** ^1^ Department of Neurology Neurology and Neurophysiology Center Vienna Austria

We read with interest the article by Hsu et al. [1] about a 47‐year‐old man with myeloid leukemia who was treated with idarubicin, cytarabine, decitabrine, and allogeneic hematopoietic stem cell transplantation. Treatment was complicated by cytomegalovirus (CMV) infection and polyneuropathy. The polyneuropathy was interpreted as Guillain‐Barré syndrome (GBS). The patient received steroids and repeated plasmapheresis with positive results [1]. The study is interesting, but some points require discussion.

First, the diagnosis of “GBS” is questionable [2]. Nerve conduction studies (NCS) showed a polyneuropathy that differs from GBS [2]. There is also no evidence of cyto‐albumine dissociation. Only IgG was elevated in the cerebrospinal fluid (CSF), which does not necessarily mean that total CSF protein was actually elevated. There was also no information indicating that the nerve roots were swollen and showed contrast enhancement on MRI with contrast medium [1]. Furthermore, no results from F‐wave studies were presented that would demonstrate an absence or delay of F‐wave responses. In addition, the latency period between the initial CMV infection and the onset of GBS symptoms was 34 days, making a causal relationship highly unlikely.

Second, it is unclear why the patient received steroids in addition to plasmapheresis [1]. Steroids are known to be ineffective in GBS [3]. They generally cause more side effects than benefits. It is equally unclear why the patient did not receive intravenous immunoglobulins (IVIG) as first‐line therapy, since these are recommended as such. Were there any contraindications for IVIG?

Third, the patient was not systematically investigated for the causes of the polyneuropathy [1]. Since he was treated with idarubicin, cytarabine, and decitabrine, and these substances can induce polyneuropathy in some cases [4], it is essential to first rule them out as the cause of the polyneuropathy. If they are not the cause, all other possible etiologies, including neoplastic and paraneoplastic neuropathy, must be excluded.

Fourth, the NCS results were not reported in detail [1]. Did they show an axonal, demyelinating, or mixed lesion? Knowing the type of electrophysiological abnormality is crucial, as treatment and prognosis can vary significantly.

In summary, before GBS can be attributed to CMV infection, the diagnosis of GBS must be confirmed and other causes must be carefully ruled out.

## Conflicts of Interest

The author declares no conflicts of interest.

## Data Availability

The data that support the findings of this study are available on request from the corresponding author. The data are not publicly available due to privacy or ethical restrictions.
